# Response to multi-generational selection under elevated [CO_2_] in two temperature regimes suggests enhanced carbon assimilation and increased reproductive output in *Brassica napus* L.

**DOI:** 10.1002/ece3.523

**Published:** 2013-03-15

**Authors:** Georg Frenck, Leon van der Linden, Teis Nørgaard Mikkelsen, Hans Brix, Rikke Bagger Jørgensen

**Affiliations:** 1 Department of Chemical and Biochemical Engineering, Technical University of Denmark, Risø CampusFrederiksborgvej 399, 4000, Roskilde, Denmark; 2 Department of Biosciences, Plant Biology, Aarhus UniversityOle Worms Allé 1, DK-8000, Aarhus C, Denmark

**Keywords:** Carbon dioxide, experimental evolution, global change, laboratory natural selection, oilseed rape, phenotypic divergence, plant-environment feedbacks

## Abstract

Functional plant traits are likely to adapt under the sustained pressure imposed by environmental changes through natural selection. Employing *Brassica napus* as a model, a multi-generational study was performed to investigate the potential trajectories of selection at elevated [CO_2_] in two different temperature regimes. To reveal phenotypic divergence at the manipulated [CO_2_] and temperature conditions, a full-factorial natural selection regime was established in a phytotron environment over the range of four generations. It is demonstrated that a directional response to selection at elevated [CO_2_] led to higher quantities of reproductive output over the range of investigated generations independent of the applied temperature regime. The increase in seed yield caused an increase in aboveground biomass. This suggests quantitative changes in the functions of carbon sequestration of plants subjected to increased levels of CO_2_ over the generational range investigated. The results of this study suggest that phenotypic divergence of plants selected under elevated atmospheric CO_2_ concentration may drive the future functions of plant productivity to be different from projections that do not incorporate selection responses of plants. This study accentuates the importance of phenotypic responses across multiple generations in relation to our understanding of biogeochemical dynamics of future ecosystems. Furthermore, the positive selection response of reproductive output under increased [CO_2_] may ameliorate depressions in plant reproductive fitness caused by higher temperatures in situations where both factors co-occur.

## Introduction

Recent and projected future alterations of environmental features cause sustained pressures on global ecosystem functioning (Fischlin et al. 2007). Important environmental parameters are currently changing at an unprecedented pace as a consequence of human activities (Bell and Collins 2008). Where the plastic response potential of a population cannot fully compensate stressful changes in environmental conditions, only evolutionary adaptation can prevent wide-ranging declines in fitness and counter the increased risk of extinction (Jump and Peñuelas 2005). Especially for sessile organisms like plants, where migration likely fails to track the speed and magnitude of environmental change, adaptive responses will be of eminent importance.

The atmospheric carbon dioxide concentration – [CO_2_] – is one of the most prominent environmental parameters currently changing. By the end of the 21st century, [CO_2_] is likely to become more than doubled compared with pre-industrial concentrations (Swart et al. 2002). [CO_2_] is also inherently coupled to, and a strong driver of global temperature regimes (Forster et al. 2007). Hence, simultaneously to the increase in [CO_2_], global average temperature is projected to increase by 1.8 to 4.0°C compared to the values at the beginning of the century (Carter et al. 2007)**.** Both [CO_2_] and temperature are documented as potent agents of directional selection in plant populations (e.g., Ward and Kelly 2004; Jump and Peñuelas 2005; Reusch and Wood 2007). Both factors affect the allocation of resources to components of plant fitness by response functions, which vary between individuals of a given population. These genotype-environment interactions are the basis for natural selection to act in intra-specific competition regimes and facilitate micro-evolutionary adaptation responses.

Adaption of phenotypes over a range of generations may lead to future biogeochemical dynamics and long-term trends of plant fitness, which are different from projections assuming evolutionary stasis (Bazzaz et al. 1995; Ward and Kelly 2004). In order to improve our understanding of future ecosystem structure, functioning, and biodiversity patterns, we need to evaluate the potential and nature of plant responses to natural selection in plants to the changes projected.

Within-generation estimates of adaptive evolution provide valuable information for species with a long generation time and/or selective processes under natural conditions. However, such estimates might lead to only incomplete and relatively limited conclusions with respect to evolutionary trajectories (Thomas and Jasienski 1996; Ward and Kelly 2004; Reusch and Wood 2007; Fuller et al. 2009). Selection experiments incorporating several generations provide a unique opportunity to evaluate and predict evolutionary responses of populations to projected changes in environments (Reusch and Wood 2007). Hence, multi-generational selection experiments will play a key role in understanding the many challenges that global environmental change will present to biodiversity and ecosystem functioning in the future (Bell and Gonzalez 2009). Until now, experimental evidence on the nature and direction of selection responses of phenotypic traits under simulated future conditions is very limited and rare. Exacerbating the limitations in our understanding of plant evolutionary responses to environmental change, the results of some of the very few studies conducted so far are biased by the applied methodology. The abundance of studies available applied artificial selection regimes to generate treatment-specific generational progress. A procedure like this may, however, interrupt the integrity of trade-offs between multiple fitness-related traits. Avoiding these methodological biases, natural selection experiments are proposed as “gold standard” to investigate the evolutionary trajectories under manipulated environmental conditions (“laboratory natural selection”, cf. Reusch and Wood 2007; Fuller et al. 2009). In natural selection experiments, populations freely adapt by fertility and mortality distributions, similar to natural conditions.

Here, we present the results of a natural selection experiment applying elevated [CO_2_] under two different temperature regimes. The response of biomass production and reproductive output to the selection regimes imposed by these environmental manipulations was investigated over a range of four generations. This study aimed to reveal if the manipulation of CO_2_ and temperature regimes is able to provide selection pressures, which are strong enough to drive phenotypic divergence over a short range of progressing generations. The experiment investigated *Brassica napus* as model species, because the species has demonstrated to complete its life cycle under laboratory and artificial light conditions within a comprehensible generation time. Explained by its crop identity, *B. napus* might experience less profound effects by the artificial growing conditions than more natural plant species, as it is already habituated to be partially detached from some ecological functions like, for example, a natural nutrient supply and interspecific interactions.

Higher plants, in their role as primary producers, crucially drive the budgets of ecologically imported cycles (e.g., carbon, nitrogen, and water). Hence, they are also major players in controlling and steering the global climate system by a multitude of feedback responses, among which the connections between carbon cycle and climate might amplify or dampen regional and global climate changes (Heimann and Reichstein 2008). It was investigated here, if phenotypic responses to the applied selective agents may point at potential uncertainties in our current assessments of future biogeochemical dynamics (e.g., carbon uptake) introduced by progressing phenotypic adaptation.

## Methods

### Environmental manipulations

**Figure 1:**
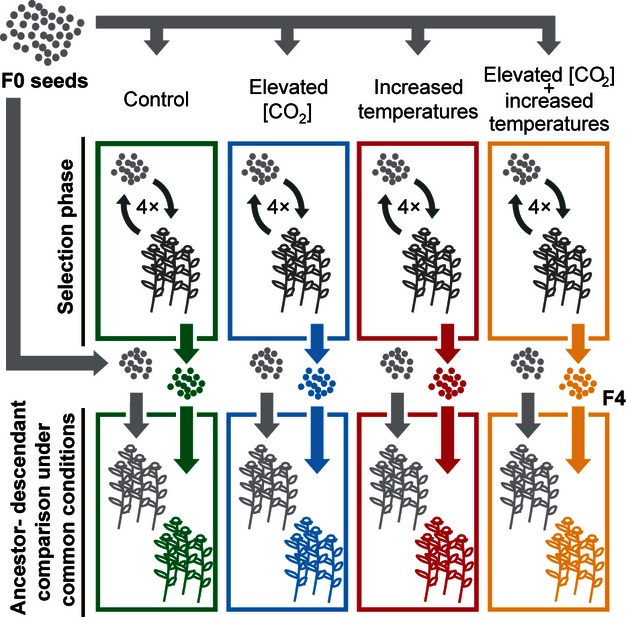
Schematic illustration of the selection experiment. *Brassica napus* populations were selected over four generations under manipulated experimental conditions, before descendent material was grown together with ancestral stock populations sharing a common environmental configuration. Experimental procedures are only represented for one of the four replicate selection linages (RSL) in each treatment.

*Brassica napus* L. plants were grown in four different environments varying in atmospheric [CO_2_] (ca. 390 vs. 650 ppm) and air temperature regimes (ca. 19/12 vs. 24/17°C) with these environmental manipulations imposed individually or in combination (Fig. 1, Table 1). Treatments were accommodated in four gastight chambers (width 6 m, depth 4 m, height 3 m) integrated in a phytotron facility at the Technical University of Denmark, DK (RERAF – Risø Environmental Risk Assessment Facility). The growth chamber system allowed individual control of air flow, light, temperature, humidity, [CO_2_], [O_3_], and watering. Twenty-eight high-pressure mercury and 14 halogen lamps (1000 W and 400 W, respectively) per chamber were used to generate a 16/8 hours day–night light regime. During daytime conditions, average photosynthetic active radiation (PAR) on top of the canopy was 520 μmol_Photons_ m^−2^ s^−1^ (measured with a Li-Cor LI-250A light meter, Li-Cor, USA). At the beginning of the daytime period, plants were watered by a drip irrigation system providing 4.4 L m^−2^ day^−1^ delivered to the individual pots. During early developmental stages, when water uptake was still low, water exceeding field capacity was allowed to drain from the soil phase. The amount of water delivered was decreased at the beginning of leaf senescence (approximately within the 3rd month after planting) in a stepwise fashion to facilitate and promote complete maturation and seed ripening. A relative humidity of 55/70% (day/night, measured with a HMP231 humidity and temperature transmitter, Vaisala, Finland) and a realistic background ozone concentration of 18.1 ppb (±15.5 ppb standard deviation, measured with the 400E ozone monitor model from Teledyne, USA) were established in the growth chambers. To prevent pathogen interference in the enclosed system (Long et al. 2006), a fungicide treatment (CANTUS®, BASF AG, Germany) was applied to all plants every second week during the first month of development. All of the manipulated environmental conditions are statistically summarized in Table 1.

**Table 1:** Summary of manipulated environmental conditions for the four treatments and temporal delayed groups of cultivation in this study: daily mean values ± their standard deviation are summarized here. Filled symbols indicate the application of elevated carbon dioxide concentration ([CO_2_]) or higher temperature (Temp.) regime; open symbols indicate lower [CO_2_] and temperature conditions

		Group 1						Group 2					
				
Treatment		[CO_2_] [ppm]		Temp. [°C]				[CO_2_] [ppm]		Temp. [°C]			
				
[CO_2_]	Temp.	Day		Day		Night		Day		Day		Night	
□	□	391.3	±20.1	18.5	±0.9	12.8	±1.0	391.8	±21.2	18.6	±0.7	13.0	±0.9
▪	□	656.5	±41.5	18.4	±1.1	12.7	±0.8	660.2	±40.6	18.3	±1.0	12.8	±0.8
□	▪	386.1	±11.3	23.4	±0.9	17.8	±0.8	389.9	±14.2	23.2	±0.9	18.0	±0.8
▪	▪	648.2	±54.9	23.5	±0.6	17.8	±0.8	650.5	±55.2	23.4	±0.6	18.0	±0.7

### Plant material and generational progress

Four parallel selection lineages were initiated in each of the four treatments. Each linage was based on one of four different accessions of *B. napus* L. (Table 2, hereafter referred to as replicate selection linage – RSL). Each of the four RSLs in every treatment consisted of 36 individuals. At the beginning of the experiment, the first generation of plants (F0) was grown from seeds until maturity and harvested. In the absence of animal pollinators, *B. napus* plants of a given RSL were gently shaken during flowering to promote within-population cross-pollination by physical contact. The different RSLs in every treatment were kept spatially separated as they were grown on individual, moveable tables.

**Table 2:** Origin, year of release, breeding organization, and distribution area of the four *Brassica napus* accessions founding the four replicate selection linages (RSL) of the experiment

Accession	Origin	Year of release	Breeder	Distribution area
Bolero	Germany	1997	Raps GbR	Southern & eastern Europe
Mary	Denmark	1978	DLF Trifolium A/S	Denmark
Mozart	Germany	1999	NPZ/Lembke	Baltic countries
Tanto	France	1990	INRA/Serasem	*No information available*

At the end of each cultivation cycle, the plant material was post-harvest processed as described below. Treatment-specific generational progress (F1, F2, F3) was established for each individual RSL-treatment combination (Fig. 1): Descendent populations of each RSL, F(g + 1), were based on seeds chosen randomly out of the pooled seed stock of its corresponding ancestor population – F(g), and were grown under the same environmental conditions as the parental population of a given RSL (Fig. 1).

During the final cycle of cultivation, the plants selected under treatment conditions for fourth generations, F4 plants of every RSL, were grown simultaneously with plants derived from the initial seed stock (F0) in every treatment. For this last cycle of cultivation, plants were grown in two batches, which were temporally displaced by 28 days (referred as groups within the description of statistical procedures). RSLs were randomly assigned to each of the two groups as a set of two – while the F0 population and F4 generation for each RSL were kept together, sharing identical environmental conditions during their development.

The control groups of this experiment were defined based on the propositions for the experimental design of laboratory natural selection experiments made by Fuller et al. (2009): (a) The control selection linages were maintained under the same demographic and environmental conditions as within the selective regimes, except for the environmental manipulations. (b) The evolved descendants were compared to the ancestral stock population. The original seed stock was maintained under long-term storage conditions throughout the duration of selection phase of the experiment (Fig. 1) implying evolutionary stasis. This procedure allowed separating directional changes unrelated to the selection regimes imposed by environmental manipulations form changes caused by the manipulated factor.

### Conditions of cultivation

Plants were cultivated in 11 L pots each filled with 4 kg of a standard sphagnum substrate (Pindstrup Substrate No. 6, Pindstrup Mosebrug A/S, Denmark). The soil substrate in each pot was supplemented with 10 g NPK fertilizer (21-3-10, Kemira Denmark A/S). A competition regime for belowground resources, including water, nutrients, and space was created for sets of four plants sharing one pot. Plant density was established to be 64 individuals per m^2^ and equally distributed over the experimental populations. The experimental populations were restricted from growing over the defined spatial margins, by a white, 4-mm meshed polyethylene net of 30-cm height, thereby also reducing lateral irradiation-induced border effects at the edges of the population.

To avoid unilateral impacts, chamber-specific biases, and to minimize confounding effects of micro-environmental variation within the chambers, the tables holding the plants and the corresponding treatments were relocated among the different chambers of the phytotron facility every week together with their corresponding treatments. At this time, the relative position of a given population within a chamber was also changed.

### Harvests

All plants were grown in the experimental environments until leaf senescence, silique, and seed ripening indicated life-cycle completion. Final harvests were performed at 109 and 116 days after planting for plants grown at elevated and lower temperature, respectively. At the final harvest, all individuals of the population were harvested as a pot-wise pooled sample, leading to nine replicate harvest samples for each experimental population. At that time, silique, stem, and leaf fractions were segregated. Siliques were dried in a force ventilated oven at 36°C (TU2, Heraeus, Germany). Leaf and stem material were first dried under ventilated room conditions before residual moisture was removed during an oven incubation at 72°C. After drying, seed and silique material were separated. Subsequently, dry weights were determined for all aboveground biomass fractions. The weight of a 100 seeds sub-sample, as enumerated by an automatic seed counter (Numigral, Sinar Technologies, UK), was determined in order to derive total seed number (Seed No.). Subsequently, mean per plant values were calculated for all response variables.

### Data analysis and statistical procedures

All statistical procedures and calculations were performed in R (version 2.11.1; R Development Core Team, 2010). Individual linear mixed-effects models were fitted for each response parameter (Pinheiro and Bates 2000). The concentration of CO_2_ and temperature regime in the experimental environments, the number of generations, and all possible interactions among those three categorical, explanatory variables were modeled as fixed effects for the initial model. Group, treatment and RSL, with a hierarchical grouping structure according to the order they are mentioned here, defined the random intercept of the initial mixed-effects model. Treatment was included as grouping variable in order to nest error within the plants sharing a particular treatment and to prevent elevation of Type I error for the effects of environmental manipulations. To elucidate the general, species-level responses across the different RSL independent from effects imposed by their distinct genetic identity, all effects were analyzed as nested into RSL.

Linear mixed-effects models were simplified in a stepwise fashion: The initial maximal model was first subjected to a backward fitting algorithm for the fixed effects. In reverse order to the degree of interactions, terms were removed in the model at a threshold of 0.05 for its lower bound *P*-value and the *P*-value for the log-likelihood ratio test between the complex and the simplified model version (cf. Tremblay 2012). Non-significant terms involved in significant higher order interactions always remained as part of the model structure. Subsequently, random slopes for the fixed effects, which remained in the most parsimonious model structure, were tested for their significance and included into the random effects definition of the model if the *P*-values for the log-likelihood ratio test between the complex and the simpler model was smaller than 0.05. The model definition finally achieved by this procedure was confirmed by a visually inspection of residual and quantile distribution according to the criteria for parametric statistical tests. The model summaries for each response variable are given in Table A1.

## Results

**Figure 2:**
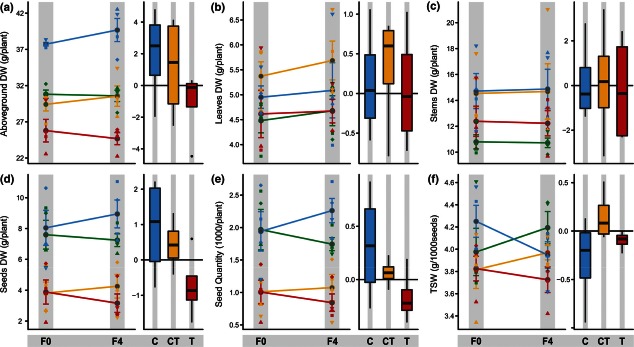
Mean values (±standard error) of biomasses fractions at final harvest determined for the four replicate selection linages of *Brassica napus* grown in a phytotron environment at the four possible combinations of two temperatures and [CO_2_] regimes (Table 1) in two generations: (a) aboveground DW, (b) Leaves DW, (c) Stems DW, (d) Seeds DW, (e) Seed Quantity, (f) thousand seed weight (TSW) F0 – ancestral stock population, F4 – fourth descendant population selected in the corresponding environments – gray-filled, round symbols connected by a line. Treatments: green – Ctrl (control), blue – elevated [CO_2_] (C), red – increased temperatures (T), orange – elevated [CO_2_] and increased temperatures (CT). Point characters of the same shape represent RSLs with the same identity. Boxplots show the difference in slopes between the trans-generational response in the given treatments and the control scenario for the four RSL: (trait value mean_F4_ – trait value mean _F0_)^C, CT, T^ – (trait value mean_F4_ – trait value mean _F0_)^Ctrl^, DW, dry weight; TSW, thousand seed weight.

All measured biomass fractions responded to the applied environmental manipulations. Final aboveground dry weight (AG DW) of *B. napus* plants was strongly affected by the CO_2_ and temperature regimes. However, the different plant organs responded differentially to the individual combinations of environmental manipulations (Fig. 2). *Brassica napus* plants increased AG DW accumulation in response to elevated [CO_2_] in the present experiment (*P* < 0.001, Fig. 2A). For the F0 generation, the [CO_2_]-related increase in AG DW was 22% and 14% in average at ambient and elevated temperature, respectively. Concurrent with the responses of total AG DW to [CO_2_], vegetative fractions of leaf and stem material increased under high [CO_2_] growing conditions (leaves: *P* = 0.0063, stems: *P* = 0.003, Fig. 2B and C). The seed fraction of *B. napus* did reveal no significant response to the applied [CO_2_] conditions.

In contrast to the response pattern seen under elevated [CO_2_], higher temperatures led to reductions in final AG DW in *B. napus* (*P* < 0.001, Fig. 2A). The decline in total AG DW under elevated temperatures was aligned with decreasing weights of the seed fraction (*P* < 0.001), while leaf and stem biomass did not reveal a response to the applied temperature regime. Under both concentrations of CO_2_, the DW of seeds in *B. napus* was significantly reduced in response to higher temperatures (Fig. 2D). The temperature-driven depression in seed number per plant was ∼50% and showing similar reductions under both [CO_2_] regimes (Fig. 2E).

As the effect of temperature on the sum of all AG biomass fractions had a larger magnitude than the promoting effect of high [CO_2_], AG DW tended to be slightly reduced when [CO_2_] and temperature were elevated concurrently compared with the corresponding control conditions (Fig. 2A).

The cultivation of *B. napus* in the experimental environments altered its responsiveness over the range of generations investigated here. As a specific response to the CO_2_ concentration, AG DW (*P* = 0.0118), DW of seeds (*P* = 0.0017), and their total number (*P* = 0.0624) were different between the ancestral stock population (F0) and the populations selected in the manipulated environments over four generations (F4, Fig. 2A, D and E). Throughout the multi-generational cultivation, the sum of accumulated AG biomass deviated to higher values from F0 to F4 in high [CO_2_] environments compared to the pattern found under ambient [CO_2_] (Fig. 2A). Under high [CO_2_] growing conditions, AG DW increased ∼4.8% and 4.1% at concurrently elevated temperatures, respectively, from F0 to F4. This CO_2_-specific response between the start (F0) and final offspring generation (F4) of the experiment significantly contrasts the decreasing of 0.7% and 3.4%, respectively, under low [CO_2_] conditions. Hence, throughout the trans-generational cultivation process, plants achieving higher final AG DW were selectively favored in environments applying the elevated [CO_2_] regime. This trans-generational alteration of the final aboveground biomass, which was found specifically in high [CO_2_] environments, was associated with a similar pattern of response in the seed fraction, whereas vegetative fractions, stems, and leaves, did not reveal any environment specific responses to the selection process. Concurrent to the pattern found for AG DW, the trans-generational alteration in the quantity of seeds produced per plant was revealed to be different according to the two CO_2_ concentrations investigated here. The absolute differentials of increase in seed yields from the F0 to the F4 generation appeared larger under lower temperature conditions (Fig. 2D and E). Hence, under elevated [CO_2_], reproductive output was increased in numbers and total weights in response to the selection regime established by elevated [CO_2_] compared to the pattern found at ambient CO_2_ concentrations (Fig. 2D and E). However, although individual seed weight was reduced from F0 to F4 in the elevated CO_2_ environment, applying the lower temperature regime, the pattern under increased temperatures was significantly different (*P* = 0.0196) and revealed an increase in the weight of individual seeds (Fig. 2F).

Within the present experiment, no differences were detected for any biomass fraction specifically to the applied temperature regimes within the generational range of this study.

## Discussion

The causes and implications of the promoting effect of [CO_2_] on plant productivity are well understood and described for numerous plant species and ecosystems (Ainsworth and Rogers 2007). In consistency with earlier greenhouse and free air CO_2_ enrichment studies (FACE, Qaderi et al. 2006; Franzaring et al. 2008), vegetative biomass in *B. napus* was increased under elevated [CO_2_]. The lack of [CO_2_]-response for seeds was earlier explained by restrictions in the sink capacity of the reproductive apparatus in *B. napus* (Reekie et al. 1998). An additional reduction in the sink potential of reproductive organs might be caused by the adverse effects of increased canopy temperatures at elevated [CO_2_] (Fuhrer 2003) as explained below. This evidence already suggests that an increase in sink strength of reproductive organs has the potential to enhance the gain in biomass productivity caused by high [CO_2_] in *B. napus*.

The decline of AG DW under elevated temperatures was associated with a decrease in reproductive productivity while other biomass fractions remained unaffected. Supra-optimal temperatures adversely affect functions of reproductive development on multiple levels (cf. Barnabás et al. 2008 for review). Consequently, increased temperatures reduce the sink strength of the reproductive apparatus in particular, while reducing the source potential of plants at the same time (Stone 2001).

The direct and negative effect of temperature on reproductive fitness demonstrates the strong selection pressure imposed by non-optimal thermal conditions, which resulted in frequent differentiations of natural populations with respect to temperature (Jump and Peñuelas 2005). In turn, rapidly changing climatic conditions have the potential to overwhelm the plastic response potential, and cause fast declines in the fitness of natural populations. If fitness cannot be maintained or restored by an adaptation to the altered conditions, the fast changes in environmental characteristics may increase the risk of local extinctions as migration rates are often likely to be insufficient for tracking the climatic conditions in which plants are currently adapted to (cf. Jump and Peñuelas 2005 for review). A study performed by Franks et al. (2007) recently provided an example of *Brassica rapa* responding adaptively to a natural multi-year drought in just a small number of generations. The evolved descendants flowered earlier to adapt their development to the drought abbreviated growing season.

In annual plants, seed production has to be maximized within a single growing season, as the recruitment of new individuals is only possible from seeds. Hence, reproductive output and fecundity become central and major components of fitness in annuals (Cheplick 2005; Hirose et al. 2005). Consequently, for an intra-specific comparison, it can be assumed that the genotype with higher reproductive quantities is likely to be the one with the higher fitness, and the one better adapted to the environment of comparison. This, however, only holds true, if no trade-offs with other fitness-related traits are implied and the competition and resource regime can be considered constant, like in the experimental setup presented here.

In our study, increased numbers and total weights of seeds produced per plant were found as a trans-generational response specific to high [CO_2_] growing conditions, when compared to the response pattern revealed under ambient CO_2_ concentration. The manipulation of the CO_2_ regime in the experimental environments significantly altered the trans-generational response pattern in reproductive output from the F0 to the F4 generation. As this was a specific response to the selective environment generated by elevated CO_2_ concentrations, seed quantities and likely associated reproductive fitness were significantly increased in the F4 generation compared with the response in environments applying lower concentrations of CO_2_. However, the pattern of the trans-generational response to elevated [CO2] for individual seed weights suggests different mechanisms for the increase in total seeds weights and numbers in the two different temperature regimes (Fig. 2F).

A positive selection response of seed production under manipulated [CO_2_] shown for *Arabidopsis thaliana* in an artificial selection regime (Ward et al. 2000) suggested that [CO_2_] provides a sufficient pressure to act on phenotypic divergence of populations. However, artificial selection regimes, where the investigator selects for a single trait which is believed to provide a fitness advantage in the wild, impose specific artifacts on the response to selection. Selection for a single, deliberately chosen trait will interfere with the genetic architecture of the trait selected for in terms of heritability, correlations, and trade-offs with other traits (Reusch and Wood 2007). Therefore, the magnitude, direction, and phenotypic integration of trait differentials revealed in artificial selection experiments might be biased and/or misleading, when they are discussed in a natural context.

A positive selection response to elevated [CO_2_], as evidenced in our study, might provide a potential to improve reproductive fitness in some plants species within a foreseeable number of generations. Thereby, fitness depressions imposed by co-emerging stressors as, in the present example, increased temperatures can partially be ameliorated by the positive effect of selection imposed by high [CO_2_], if the pattern seen in this study is representative for natural conditions.

Our results show no phenotypic divergence of the *B. napus* linages in response to the temperature manipulations. We hypothesize that the selection regime imposed by the thermal conditions in the experiment was stabilizing. This stabilizing selection might have been result of the limited temperature variability in the experimental environments. Spatial and temporal environmental variation is an important driver to maintain genotypic and consequently phenotypic diversity of populations by creating alternating selection regimes (Gutschick and Bassirirad 2003; Jump et al. 2009). Hence, it appears plausible that in the absence of environmental heterogeneity and variability, like in the experimental system presented here, populations will become more “streamlined”.

The overall increase in AG DW from F0 to F4 suggests a trend to higher biomass productivity of the plants selected in high [CO_2_] environments over the generational range of this study. Our results suggest a likely qualitative alteration of plant-mediated carbon accumulation as a trans-generational response to the manipulated CO_2_ levels in the experiment. Today, the abundance of projections considering the long-term trajectory of plant-atmosphere feedbacks assume plant responses to be stable over the predicted timescales. However, our study shows that important functions related to carbon assimilation and plant fitness might change in response to selection pressure imposed by increasing [CO_2_]. Therefore, the presented experimental evidence reinforces earlier propositions that long-term projections regarding future biogeochemical dynamics may lead to incomplete or incorrect conclusions, if they neglect likely responses to changed environments, which occur over progressing generations or fail to incorporate these effects (Bazzaz et al. 1995; Ward and Kelly 2004). Wieneke et al. (2004) detected a weak adaptive response toward increased biomass production in *Sanguisorba minor* under increased concentrations of [CO_2_]. However, for *Sanguisorba minor,* embryological data document gametophytic apomixes (Dickinson et al. 2007), implying that the establishment of any treatment-specific offspring during their experiment is speculative and requires further clarification, for example, by genetic data.

A potential explanation of the rapid phenotypic divergence seen in the experimental populations of the present experiment as a trans-generational response to the environmental manipulations may be provided by the selection of beneficial genotypic configurations. Fast genetic adaptation responses can occur within a small number of subsequent generations (Barrett and Schluter 2008). However, non-genetic inheritance mechanisms may offer an alternative explanation for the fast and specific responses to the different selection scenarios in this study (Bonduriansky 2012). Even though this study cannot fully resolve if the underlying molecular mechanism of the trans-generational response is DNA-sequence based or epigenetic (including parental/maternal), it clearly suggests a selection response as fitness differentials of the experimental populations were revealed over the range of generations investigated. We further argue that the separation of genetic and non-genetic causes for the observed divergence is unsubstantial for the discussion of functional differentiation of plant phenotypes in a [CO_2_]-context. Both, DNA-sequence based and non-genetic pathways provide heritable phenotypic adaptation to environmental change, which is persistent over multiple generations and only differ in the speed of the response. However, fitness enhancing, maternal environmental effects are unlikely to produce phenotypic divergence in response to environmental conditions experienced by all individuals of a given population, like, for example, elevated CO_2_ concentrations. In such scenarios, natural selection is expected to be the primary force to define adaptive responses of a given population (Galloway 2005).

The relatively small number of individuals in the present experiment might have imposed a genetic bottleneck to the experimental populations. Such bottlenecks have been reported to increase additive genetic variance, leading to faster selection responses as in more diverse populations as high levels of genetic diversity may dampen the immediate response to selection (Edwards and Lamkey 2003). Therefore, our experiment is likely to have detected an accelerated response to selection imposed by the environmental manipulations. Rapid adaptation responses strongly depend on the pool of immediately available alleles, rather than beneficial mutation events (Barrett and Schluter 2008). Consequently, the rate at which recombination generates new allelic combinations and all parameters which affect the amount of recombination events per unit time define the potential speed of adaptation. Hence, the rates of micro-evolution will, for every natural population, depend on a unique and specific set of multiple abiotic and biotic parameters (e.g., habitat structure, gene flow, generation time, breeding strategy, self-compatibility, etc.), making general predictions virtually impossible (Bone and Farres 2001).

In this study, the potential of plants to adjust phenotypes to rapid environmental change over a range of progressing generations beyond the initial plastic response was clearly shown by the significant and specific trans-generational response. Also, the direction of this response under elevated [CO_2_] conditions was clearly indicated for a higher plant species model. The chances of a seed of any given mother plant to be selected for the next generation in the experiment was proportional to the mother plants relative contribution to the seed output of the entire population. Hence, selection by competitive exclusion, similar to natural conditions, was warranted on the basis of quantity of reproductive output. Irrespective to the underlying molecular basis of the phenotypic selection response, we were able to reveal a dimension of plant-environment feedbacks, which is currently insufficiently investigated and described for the response of plants to future CO_2_ concentrations. The results of this study reinforce that fast phenotypic divergence in response to selection imposed by anthropogenic environmental change can alter fitness distributions in plant populations over a short range of generations and imply that future ecological dynamics might differ from projections that do not incorporate adaptive change in plants. Therefore, this experiment demonstrates the importance of responses across several generations in relation to our understanding of future plant-environment feedbacks. The results of this study ask for a broader scientific approach and further investigations in order to define the magnitude of plant responses to rapid environmental change in a multi-generational and evolutionary context.

## Appendix

**Table A1:** Analysis of variance (ANOVA) results for linear mixed-effects models integrating the independent and all interactive effects (×) of [CO_2_] (C), temperature (T), and generation (G) on finally achieved aboveground biomass fractions for four replicate selection lines of *Brassica napus* grown under two temperature regimes and two CO_2_ concentrations (Table 1) in a phytotron environment. Only effects, which explain a significant amount of variation in the data and/or are included in the most parsimonious model, are shown here: *df*, degrees of freedom; den. *df,* degrees of freedom for the denominator; upper and lower bound den. *df* and *P*-values shown as extracted with the method provided by Tremblay (2012); *P*-values below 0.05 are presented in bold with standard significance indices; DW, dry weight

Response	Source	*df*	*F*-value	Upper den. *df*	Upper *P-*value	Lower den. *df*	Lower *P-*value
Aboveground DW	[CO_2_] (C)	1	31.3071	283	**0*****	257	**0*****
	Temperature (T)	1	40.2058	283	**0*****	257	**0*****
	Generation (G)	1	0.7873	283	0.3757	257	0.3758
	C × G	1	6.431	283	**0.0118***	257	**0.0118***
Leaves DW	[CO_2_]	1	7.5758	286	**0.0063****	260	**0.0063****
Stems DW	[CO_2_]	1	8.9545	286	**0.003****	260	**0.003****
Seeds DW	[CO_2_]	1	1.0453	283	0.3075	257	0.3075
	Temperature	1	32.7189	283	**0*****	257	**0*****
	Generation	1	0.0578	283	0.8102	257	0.8102
	C × G	1	10.024	283	**0.0017****	257	**0.0017****
Seed number	[CO_2_]	1	3.4348	283	0.0649	231	0.0651
	Temperature	1	38.5349	283	**0*****	231	**0*****
	Generation	1	0.0013	283	0.9715	231	0.9715
	C × G	1	3.5074	283	*0.0621*.	231	*0.0624*.
1000 Seed weight	[CO_2_]	1	0.3253	280	0.5689	228	0.569
	Temperature	1	5.4091	280	**0.0207***	228	**0.0209***
	Generation	1	0.0197	280	0.8884	228	0.8884
	C × T	1	0.4343	280	0.5104	228	0.5105
	C × G	1	0.4083	280	0.5234	228	0.5235
	T × G	1	0.1296	280	0.7191	228	0.7192
	C × T × G	1	5.5216	280	**0.0195***	228	**0.0196***
